# Key Performance Indicators Predictive of Success in Soccer: A Comprehensive Analysis of the Greek Soccer League

**DOI:** 10.3390/jfmk9020107

**Published:** 2024-06-17

**Authors:** Andreas Stafylidis, Athanasios Mandroukas, Yiannis Michailidis, Lazaros Vardakis, Ioannis Metaxas, Angelos E. Kyranoudis, Thomas I. Metaxas

**Affiliations:** 1 Laboratory of Evaluation of Human Biological Performance, Department of Physical Education and Sports Sciences, Aristotle University of Thessaloniki, 541 24 Thessaloniki, Greece; andreas.stafylidis@gmail.com (A.S.); thanmandrou@hotmail.com (A.M.); vardakis@live.com (L.V.); aggelos_1970@yahoo.gr (A.E.K.); tommet@phed.auth.gr (T.I.M.); 2 Department of Physical Education and Sports Sciences, Aristotle University of Thessaloniki, 621 00 Serres, Greece; metaxasi@phed-sr.auth.gr

**Keywords:** performance analysis, binary logistic regression, success prediction, soccer, match-performance analysis, football

## Abstract

Previous research emphasizes the significance of key performance metrics in determining match outcomes. The purpose of this study is to enhance the understanding of success in professional soccer by analyzing the relationship between match outcomes (win, lose, draw) and various Performance Indicators extracted from the Greek soccer league, as well as to develop a regression model of success in soccer. The sample consisted of all 91 matches from the first round of the 2020–2021 season of the Greek Football League. Utilizing Kruskal–Wallis tests, significant differences were found in goals scored, shots, and shots on target, ball possession, passing metrics, touches in the penalty area, and average shot distance (*p* < 0.05), with winning teams having demonstrated superior performance metrics. Moreover, winning teams engaged more in positional attacks and counterattacks with shots (*p* < 0.05). The binary logistic regression model applied to predict match outcomes identified shots on target, counterattacks, passes metrics, offensive duels and set pieces (penalties, free kicks) as key factors influencing the likelihood of winning (*p* < 0.05). These findings collectively highlight the importance of effective offensive play, including goal scoring, shooting accuracy, and ball possession, in determining the outcomes of soccer matches, with the regression model offering a nuanced understanding of these relationships.

## 1. Introduction

Over the years, several studies have sought to decipher the relationship between playing styles and statistical indicators in soccer in various leagues and competitions [1,2,3,4,5,6,7,8,9,10,11,12,13,14]. Specifically, success in soccer has been strongly related to the ability of the coaching staff to observe, interpret, and improve key performance indicators and the team’s tactical behavior through interventions during the game [15,16]. In general, it is emphasized that performance analysis methods are valuable in enhancing the understanding of athletic performance by providing detailed and objective insights into players’ strengths and areas for development, enabling targeted interventions for the coaching staff [17]. Moreover, recent research highlights the significance of artificial intelligence and factor analysis in understanding playing styles in soccer [18].

The ability to score goals and prevent goal-scoring opportunities from the opponent has long been the focus of tactical discussions. While goals per se might be the ultimate aim, the road to victory is paved with a more nuanced statistic: scoring efficiency [19]. Broich et al. [19] elucidated that the quality of shots, as indicated by goal efficiency (ratio of goals scored/shots taken), holds greater significance and takes precedence over shot quantity when determining victory in a soccer match. Regarding shots on target and goals during the 2012 European Championship, it was indicated that 89% of the goals were scored from within the penalty area, while 65% of the shots from outside were saved by the goalkeepers [20]. Similar studies of the Premier League and Bundesliga during the 2012–2013 season reported that both distance and angle of the shots taken have a significant impact on the calculation of xG (Expected Goals) [21].

Regarding passing metrics and, specifically, the effectiveness of passing, it is revealed that short sequences facilitate a dynamic possession game, while longer sequences are more effective in creating shots and goal opportunities [22,23]. Other studies [3,11,12,24,25,26,27,28,29,30] provide a comprehensive examination of scoring chances, noting the significance of organized attacks, counterattacks, and set pieces. Furthermore, several studies [25,27,31,32,33,34] underscore the tactical relevance of crosses, particularly in challenging match situations, such as the utility of crosses in generating goal-scoring opportunities, with research [35,36] illustrating their effectiveness in different leagues and field locations. Moreover, several researchers [14,37,38,39,40,41,42,43,44,45,46] highlight the relationship between ball possession, passing accuracy, and game metrics such as goals and shots. These studies present a complex picture of the variables influencing success in professional soccer, highlighting the need for continual adaptation in coaching and player performance.

The application of regression analyses in soccer research has been pivotal in enhancing the understanding of the game’s strategic and tactical aspects, its effectiveness, and factors influencing match outcomes. Previous research [47] employed linear regression and factor analysis to identify key playing styles in soccer, such as possession play, set pieces, and counterattacks. Similarly, a multilevel logistic regression was applied [29] to analyze team possessions in the Premier League, highlighting the effectiveness of counterattacks and home advantage. Logistic regression analyses focused on the effectiveness of counterattacks and passing strategies [48,49,50] revealed that counterattacks were more effective against imbalanced defenses and that successful teams performed fewer passes and dribbles but completed more successful passes and shots. This suggests that a direct style of play is more successful. Other studies [51,52] explored the prediction of home team win probabilities in European soccer leagues using a regression model, emphasizing the significance of defensive performance in match outcomes. A previous study [1] investigated factors influencing ball recovery in elite soccer, identifying key variables such as match location, status, and quality of opposition, which underscored the proactive defensive strategies of higher-ranked teams. When the performance of a professional soccer team over three seasons was compared [53], it was found that successful performances were associated with fewer attempted and completed passes and more effective shooting. Kite and Nevill [53] also concluded that a more direct style of play, with fewer passes and more shots on target, benefited the team.

The importance of regression analysis in soccer research is evident. Although numerous studies have explored the differences between match outcomes (win, draw, lose), the proportion of research conducted to construct regression models that indicate the relationship between performance indicators and the prediction of match outcomes is disproportionately small. In this context, this study aimed to (a) investigate the relationship between various factors and match outcomes (win, lose, draw) and offensive and defensive play, and set pieces and (b) apply a binary regression model (win—no win) to determine if there are statistically significant key factors for teams’ success in the Greek soccer league.

## 2. Materials and Methods

### 2.1. Sample

The sample consisted of all matches of the first round (N = 91) of the first division of the Greek Football League (Super League Interwetten) during the 2020–2021 season, in which 14 teams participated.

### 2.2. Data Collection and Analysis Procedures—Analysis of Matches

All matches and statistical performance indicators presented and analyzed in this study were collected from the Wyscout platform website (https://wyscout.com, accessed on 1 June 2020) through Hudl, a platform for collecting match data by expert video analysts. Two UEFA A licensed coaches and one UEFA Pro coach were involved in the data collection and assessment process from August 2020 until August 2021. All variables’ definitions used for this study were defined in the platform’s glossaries (Wyscout Glossary, https://dataglossary.wyscout.com, accessed on 1 June 2020). For example, Pressing intensity (PPDA) quantifies high press intensity by calculating the ratio of opponent passes to defensive actions within the final 60% of the field; smart passes are creative and penetrative passes that break the opposition’s defensive lines; deep completed crosses are targeted to the zone within 20 m of the opponent’s goal; passes into the final third originate outside the final third and the next ball touch occurs within it; progressive passes are forward passes that advance the team significantly closer to the opponent’s goal, Average pass length measures the average length of passes made by a team or player during a match and average shot distance is the mean distance (m) from a team’s shots to the opponent’s goal, calculated from all shots taken by the team during a match (Wyscout Glossary, https://dataglossary.wyscout.com, accessed on 1 June 2020).

According to researchers [54], the reliability index of the Wyscout platform was 0.70, an index considered satisfactory for the analysis and evaluation of the performance of soccer players through the machine learning used by the platform. Researchers have used this platform in the past in similar research procedures [11,12,25,27,54,55,56,57,58,59,60].

### 2.3. Statistical Analysis

The data of the present study were analyzed using IBM SPSS Statistics for Windows, Version 25.0, Armonk, NY, USA, IBM Corp. [61]. Effect size (ES) was calculated according to Cohen’s criteria [62,63], and the calculation of statistical power and ES was performed with the software G*Power: Statistical Power Analyses for Windows, Version 3.1.9.7 [64,65]. Regarding the ES, the magnitude of coefficient η^2^ was evaluated in the following ranges: η^2^ = 0.01–0.06 (small effect), η^2^ = 0.06–0.14 (moderate effect), and η^2^ > 0.14 (large effect). At the level of descriptive statistics, the following were calculated: mean (M), standard deviation (SD), and frequencies for all performance indicators. Non-parametric statistical tests, specifically the Kruskal–Wallis and Mann–Whitney U tests, were applied for the comparisons between performance indicators and matches’ outcome (win/lose/draw), and in the event of a significant difference, Mann–Whitney U-tests using the Bonferroni correction were employed.

The second objective of the statistical analysis was to determine the factors that significantly predict the outcome of the match. To achieve this, a generalized linear model used for predicting binary outcomes, was utilized. The logistic regression analysis used the binary match outcome (Win versus Draw/Lose) as the dependent variable, as in previous studies [50,53]. In order to evaluate the model’s predictive capacity, we used two pseudo-R-squared values: Cox and Snell R^2^ and Nagelkerke R^2^. The −2 Log likelihood was employed as a goodness-of-fit test to assess how well the model fits the data. These measures allowed us to examine both the explained variance and the overall fit of the binary logistic regression model. The Omnibus Test of Model Coefficients was used to assess the model fit. Several predictor variables were tested for their effect on the match outcome. These predictors were evaluated based on the Wald chi-square test. The stepwise logistic regression was conducted to achieve a model that adequately predicts the match outcome (Win versus Draw/Lose), adding variables based on their ability to improve the model’s fit. The level of statistical significance was set up at *p* ≤ 0.05.

## 3. Results

The analysis of offensive play indicated significant differences across various metrics among winning, drawing, and losing teams (Table 1). The Kruskal–Wallis tests revealed that winning teams scored significantly more goals (M = 2.09, SD = 1.07) compared to drawing (M = 1.15, SD = 0.82) or losing ones (M = 0.46, SD = 0.81), with a notable effect size (H = 81.6877, *p* < 0.001, η^2^ = 0.451). Furthermore, regarding the number of shots and shots on target, winning teams exhibited a higher average (M = 11.29, SD = 4.73 for shots; M = 5.27, SD = 2.80 for shots on target), with significant differences (H = 18.5162 for shots and H = 42.488 for shots on target, both *p* < 0.001). The ratio of shots/shots on target also differed significantly across outcomes (H = 21.337, *p* < 0.001, η^2^ = 0.117).

Moreover, the average shot distance was shorter for winning teams (M = 16.89, SD = 2.76) compared to losing ones (M = 18.65, SD = 3.04, H = 11.5716, *p* = 0.003, η^2^ = 0.063). Ball possession and passing metrics were also higher in winning teams (ball possession: M = 52.65, SD = 9.11, accurate passes: M = 343.09, SD = 95.75) compared to losing ones (H = 11.244 for ball possession and H = 8.775 for accurate passes, *p* < 0.05).

Additionally, winning teams engaged in more positional attacks (M = 28.45, SD = 8.90) and counterattacks with shots (M = 2.90, SD = 2.76) compared to their losing counterparts (H = 7.848 for positional attacks and H = 9.178 for counterattacks, *p* < 0.05). Touches in the penalty area were also notably higher for winning teams (M = 16.75, SD = 7.88, H = 15.552, *p* < 0.001, η^2^ = 0.085).

Significant differences were also noted regarding the number of set pieces with shots (Table 2), with winning teams averaging 4.13 (SD = 3.88), which was higher than the drawing (M = 3.19, SD = 2.23) and the losing ones (M = 2.75, SD = 1.80, H = 8.160, *p* = 0.017, η^2^ = 0.045). Similarly, corners and corners resulting in shots were significantly different across outcomes. Winning teams had more corners (M = 5.15, SD = 3.37) and corners with shots (M = 1.72, SD = 1.46) compared to teams that drew or lost, H = 7.524, *p* = 0.023, η^2^ = 0.041 for corners and H = 8.170, *p* = 0.017, η^2^ = 0.045 for corners with shots. Penalties and penalties converted also varied significantly. Winning teams had a higher average of penalties (M = 0.40, SD = 0.55) and penalties converted (M = 0.38, SD = 40.52%) compared to the drawing (M = 0.15, SD = 0.41) and the losing ones (M = 0.07, SD = 0.26, H = 19.080, *p* = 0.001, η^2^ = 0.105). Additionally, significant differences were found regarding free kicks (H = 7.618, *p* = 0.022, η^2^ = 0.042). However, no significant differences were found in the number of fouls, free kicks resulting in shots, and offsides across different match outcomes.

Regarding defensive play, the most notable finding concerned goals conceded (Table 3). Winning teams conceded fewer goals (M = 0.46, SD = 0.81) compared to the drawing (M = 1.15, SD = 0.82) and the losing ones (M = 2.09, SD = 1.07), (H = 81.6877, *p* < 0.001, η^2^ = 0.451). However, there were no statistically significant differences for other defensive metrics such as number of losses (H = 0.9708, *p* = 0.615, η^2^ = 0.005), recoveries (H = 3.9588, *p* = 0.138, η^2^ = 0.021), duels (H = 4.3554, *p* = 0.113, η^2^ = 0.024), aerial duels (H = 3.7104, *p* = 0.156, η^2^ = 0.020) and interceptions (H = 4.6712, *p* = 0.097, η^2^ = 0.025). Similarly, metrics like offensive and defensive duels, as well as the percentage of duels won, showed no significant differences. In summary, while the number of goals conceded varied significantly according to match outcomes, other defensive metrics such as losses, recoveries, duels, and tackles did not show significant differences.

The logistic regression model was applied to predict the outcome of soccer matches as either a win or no-win (draw/lose). The model summary, as shown in Table 4, indicates that the final model achieved a −2 Log likelihood of 144.261. The Cox and Snell R^2^ and Nagelkerke R^2^ values were 0.400 and 0.549, respectively, suggesting a moderate fit of the model. Notably, the model was able to classify 83.5% of the cases correctly.

The data were explored using several logistic regression models, with the final model identifying, as shown in Table 4, the key predictors of match outcomes. The variable ‘Shots on target’ had a significant positive effect on the likelihood of winning (B = 0.526, S.E. = 0.119, Wald = 19.560, *p* < 0.001), with an odds ratio of 1.693. ‘Losses (Medium)’ was marginally non-significant (B = 0.046, S.E. = 0.023, Wald = 3.797, *p* = 0.051). Counterattacks significantly increased the likelihood of winning (B = 0.270, S.E. = 0.117, Wald = 5.305, *p* = 0.021), with an odds ratio of 1.310. Conversely, ‘Free kicks’ had a significantly negative effect (B = −0.211, S.E. = 0.061, Wald = 12.142, *p* < 0.001), indicating that an increase in free kicks is associated with a lower likelihood of winning. ‘Penalties converted’ had a substantial positive impact (B = 1.953, S.E. = 0.492, Wald = 15.745, *p* < 0.001), with an odds ratio of 7.051. The variables ‘Offensive duels won’ and ‘Back passes (accurate)’ also showed significant effects, with ‘Offensive duels won’ decreasing the likelihood of winning (B = −0.081, S.E. = 0.034, Wald = 5.629, *p* = 0.018) and ‘Back passes (accurate)’ also showing a negative association (B = −0.223, S.E. = 0.104, Wald = 4.580, *p* = 0.032). ‘Smart passes’ were positively associated with winning (B = 0.168, S.E. = 0.069, Wald = 5.910, *p* = 0.015). In summary, the binary logistic regression model identified several key factors that significantly impact the likelihood of winning in soccer. Foremost among these are ‘Shots on Target’, ‘Counterattacks’, and ‘Penalties Converted’, each playing a pivotal role in enhancing a team’s chances of winning.

## 4. Discussion

This study presents a comprehensive analysis of the relationship between various performance indicators and match outcomes in the Greek soccer league, offering a deeper understanding of the factors influencing soccer success. Specifically, the analysis revealed that winning teams typically scored an average of 2.09 goals per game, significantly higher than drawing or losing ones. This aligns with previous studies [19], as well as with other researchers [11,12], who emphasized scoring efficiency over shot quantity. As indicated by goal efficiency, the quality of shots is paramount in determining victory, supporting our observation that effective shooting is crucial for success in soccer matches.

Regarding shots on target, winning teams also had a higher average (5.27 per game). This finding is consistent with similar research [20], where it was found that most goals during the 2012 European Championship were scored from within the penalty area, suggesting that strategic positioning and accuracy are more critical than the sheer volume of shots attempted.

Furthermore, this study partially highlights the effectiveness of short passing sequences, with winning teams averaging 4.01 passes per possession, aligned with other researchers [22], who mentioned that shorter passing sequences are common and effective in creating dynamic possession games. However, our findings suggest that longer sequences could also be effective in certain contexts, echoing similar findings [23] on the strategic use of teams’ possessions. A preference for combinative attacks among high-ranked teams is also indicated in the present study, which is in line with similar analyses of other European championships [26,29]. This preference underscores the adaptability of teams based on their competitive standing [11,12,24,34]. These findings collectively underscore the importance of effective offensive play, including goal scoring, shooting accuracy, and ball possession, as explored by other researchers [25,27,31,32,42,60,66].

Our findings highlight that set plays, particularly those leading to shots, penalties and corners, could play a significant role in soccer success since winning teams generally performed better in these aspects. The observed relationship between the frequency of set pieces with shots and winning outcomes resonates with the findings of several other researchers [3,9,15,40,67], who have highlighted the critical role of performance indicators, including set pieces, in influencing match results. Furthermore, the notable differences in penalties and their conversion rates among winning teams in this study align with the arguments presented above [19]. The higher percentage of penalties converted by winning teams underscores the critical role of efficient scoring opportunities in soccer success, suggesting that the ability to capitalize on these chances can be a decisive factor in match outcomes.

Regarding defensive metrics, the significant difference in goals conceded among winning, drawing, and losing teams is a finding of considerable interest. This observation is in line with the tactical discussions emphasized by the researchers [15,23,28,68], who have explored the impact of defensive strategies on game outcomes. In this study, other defensive metrics did not show significant differences across match outcomes, echoing the general importance of a well-structured defensive strategy [30] for all teams, regardless of their ranking. While the effectiveness in preventing goals is a distinguishing factor in winning matches, other aspects of defensive play are consistently executed across different match outcomes.

The application of logistic regression analysis in this study to predict soccer match outcomes as either a win or no-win (draw/lose) provides a compelling insight into the multifaceted nature of soccer tactics and strategies. The model’s ability to correctly classify 83.5% of the cases underscores its efficacy in capturing the complexities inherent in soccer match outcomes. A key finding from this study is the significant positive effect of ‘Shots on target’ on the likelihood of winning. This aligns with the broader narrative in soccer analytics regarding the importance of creating and capitalizing on scoring opportunities, i.e., the critical role of effective offensive strategies in determining match outcomes [26,29,47]. This study also reveals the tactical significance of ‘Counterattacks’ in terms of the likelihood of winning. This finding echoes previous insights [48,49,69,70] that emphasized the effectiveness of counterattacks, particularly against imbalanced defenses. It suggests that teams exploiting quick transition opportunities are more likely to succeed. Furthermore, the substantial positive impact of ‘Penalties converted’ on winning outcomes underscores the importance of efficiency in scoring, particularly in high-value opportunities like penalties. This aspect of the game is often highlighted in studies that focus on set pieces. Conversely, the negative association of ‘Free kicks’ and ‘Back passes’ with winning outcomes suggests a potential strategic drawback in over-relying on this style of play. This could indicate that certain defensive or conservative strategies might not be as conducive to winning, adding a layer of complexity to the ongoing discourse on the balance between offensive and defensive play in soccer. Additionally, the positive association of ‘Smart passes’ with winning outcomes highlights the importance of intelligent ball distribution and strategic playmaking. Identifying these variables as key factors influencing the likelihood of winning aligns with previous findings [29,47].

While this study provides valuable insights into the analyzed matches, it is important to acknowledge its limitations. Firstly, the study’s scope is limited to the first round and not the whole season of a championship, and secondly, factors such as home advantage and in-game tactical behavior were not investigated.

## 5. Conclusions

This analysis revealed that winning teams had a higher number in metrics such as goals scored, shots on target, and passing accuracy, emphasizing the importance of effective shooting and ball possession in achieving successful outcomes. This analysis also highlighted the significance of counterattacks and successful offensive duels, suggesting that strategies focusing on quick transitions and offensive one-on-one situations can be beneficial. Additionally, winning teams tend to excel in set plays (e.g., corners, penalties, free kicks), suggesting that a focused approach to enhancing set-piece strategies could also be crucial. The logistic regression model further enriches these insights by identifying key factors influencing the likelihood of winning. An increase in shots on target is related to a higher probability of winning, emphasizing the need for strategies that create more shooting opportunities and improve shooting accuracy. Effective counterattacks also increased the chances of winning, suggesting that teams should train in quick transition play and exploit opportunities during counterattacks. Conversely, the logistic regression model indicated that an increase in free kicks and back passes is associated with a lower likelihood of winning, possibly pointing towards a need for more direct play and reducing unnecessary free kicks.

These findings provide valuable guidance for coaches, players, and performance analysts in their strategic planning and decision-making, contributing to the development of effective soccer strategies, specifically indicating that training should focus on enhancing shooting accuracy, pass completion, and developing tactics that leverage counterattacks to increase the likelihood of winning matches.

## Figures and Tables

**Table 1:** Offensive play metrics and their impact on match outcomes (win, draw, lose).

Variable	Win (*n* = 65)	Draw (*n* = 52)	Lose (*n* = 65)	H	*p*	η^2^
	M ± SD	M ± SD	M ± SD			
Goals scored	2.09 ± 1.07	1.15 ± 0.82	0.46 ± 0.81	81.687	0.001 *^†‡^	0.451
Shots	11.29± 4.73	9.57 ± 3.95	8.01 ± 3.45	18.516	0.001 *^†^	0.102
Shots on target	5.27 ± 2.80	3.69 ± 2.05	2.49 ± 1.67	42.488	0.001 *^†‡^	0.234
Shots/Shots on target (ratio)	44.68 ± 14.52	38.73 ± 16.91	31.53 ± 16.65	21.337	0.001 *^†^	0.117
Shots from outside the penalty area	4.82 ± 5.53	3.90 ± 2.04	3.40 ± 1.81	3.322	0.190	0.018
Shots from outside the penalty area on target	1.29 ± 1.33	0.96 ± 1.06	0.76 ± 0.93	6.076	0.048 ^†^	0.033
Average shot distance	16.89 ± 2.76	17.51 ± 3.64	18.65 ± 3.04	11.571	0.003 ^†^	0.063
Ball possession	52.65 ± 9.11	50.00 ± 8.73	47.51 ± 8.33	11.244	0.004 ^†^	0.062
Average passes per possession	4.01 ± 1.93	3.35 ± 0.84	3.31 ± 0.72	9.942	0.007 ^†‡^	0.054
Positional attacks	28.45 ± 8.90	26.36 ± 8.82	24.21 ± 8.08	7.848	0.020 ^†^	0.043
Positional attacks with shots	6.15 ± 3.81	4.57 ± 2.47	4.36 ± 2.48	8.116	0.017 ^†^	0.044
Counterattacks	2.90 ± 2.76	2.26 ± 1.51	1.70 ± 1.63	9.178	0.010 ^†^	0.050
Counterattacks with shots	1.03 ± 1.52	0.71 ± 0.75	0.32 ± 0.64	15.683	0.001 *^†^	0.086
Penalty area entries: runs	3.40 ± 2.60	2.67 ± 1.84	2.18 ± 1.81	11.239	0.004 ^†^	0.062
Penalty area entries: crosses	8.38 ± 4.51	9.05 ± 5.48	8.12 ± 3.87	0.275	0.871 ^†^	0.001
Touches in the penalty area	16.75 ± 7.88	13.94 ± 7.00	11.63 ± 5.84	15.552	0.001 ^†^	0.085
Passes	409.35 ± 107.74	369.80 ± 90.74	360.87 ± 75.98	8.775	0.012 ^†^	0.048
Passes accurate	343.09 ± 95.75	297.02 ± 85.73	289.68 ± 70.70	8.775	0.012 ^†‡^	0.048
Passes accurate (%)	83.61 ± 5.30	80.31 ± 4.74	80.27 ± 3.88	11.126	0.004 ^†‡^	0.061
Deep completed passes	7.23 ± 4.81	5.17 ± 3.43	3.75 ± 2.48	8.461	0.015 *^†‡^	0.046
Forward passes	141.57 ± 30.12	132.31 ± 21.37	133.31 ± 20.06	22.764	0.001 ^‡^	0.125
Forward passes accurate	105.69 ± 25.76	92.79 ± 19.78	93.48 ± 17.85	6.094	0.047 ^†‡^	0.033
Back passes	59.99 ± 15.08	50.06 ± 12.95	51.15 ± 13.20	8.506	0.014 ^†‡^	0.046
Back passes accurate	55.17 ± 14.70	46.65 ± 12.73	47.45 ± 12.46	15.115	0.001^†‡^	0.083
Lateral passes	150.85 ± 54.28	135.38 ± 53.77	124.80 ± 44.49	11.795	0.003 ^†^	0.065
Lateral passes accurate	134.66 ± 50.74	116.73 ± 50.24	108.43 ± 41.37	7.737	0.021 ^†‡^	0.042
Long passes	46.98 ± 11.50	50.88 ± 9.18	48.06 ± 8.53	9.365	0.009 ^‡^	0.051
Long passes accurate	27.29 ± 7.58	28.85 ± 6.99	27.46 ± 6.25	5.354	0.069	0.029
Passes to final third	52.39 ± 13.35	51.06 ± 13.70	46.15 ± 12.82	2.073	0.355	0.011
Passes to final third accurate	37.12 ± 12.62	33.63 ± 12.04	29.80 ± 9.62	7.123	0.028 ^†^	0.039
Progressive passes	72.94 ± 12.89	69.35 ± 13.34	66.51 ± 12.77	11.750	0.003 ^†^	0.064
Progressive passes accurate	54.78 ± 14.44	50.42 ± 14.00	47.14 ± 12.49	8.046	0.018 ^†^	0.044
Smart passes	6.65 ± 8.87	3.77 ± 2.74	3.89 ± 2.43	9.225	0.010 ^†‡^	0.050
Smart passes accurate	2.40 ± 2.03	1.48 ± 1.59	1.48 ± 1.30	11.232	0.004 ^†‡^	0.062
Average pass length	19.84 ± 1.32	20.58 ± 1.12	20.06 ± 1.22	9.299	0.010 *^‡^	0.051
Crosses	15.95 ± 12.38	14.63 ± 7.72	12.71 ± 5.94	4.193	0.120	0.023
Crosses accurate	4.77 ± 2.89	4.88 ± 3.34	3.82 ± 2.38	4.439	0.100	0.024
Deep completed crosses	5.14 ± 4.78	4.71 ± 3.24	4.23 ± 2.59	1.080	0.580	0.005
Penalty area entries: runs, crosses	21.66 ± 8.64	20.19 ± 8.69	18.18 ± 6.33	5.506	0.060	0.030
Penalty area entries: crosses	8.38 ± 4.52	9.06 ± 5.49	8.12 ± 3.88	0.275	0.870	0.001
Pressing intensity (PPDA)	8.26 ± 3.16	8.98 ± 3.47	9.94 ± 4.44	5.827	0.050 ^†^	0.032

Note: M ± SD—Mean ± Standard Deviation, H—Kruskal–Wallis H statistic, *p*—*p*-value, η^2^—Eta Squared. * Lose ≠ Draw (*p* < 0.05)|^†^ Lose ≠ Win (*p* < 0.05)|^‡^ Draw ≠ Win (*p* < 0.05).

**Table 2:** Set play metrics and their impact on match outcomes (win, draw, lose).

Variable	Win (*n* = 65)	Draw (*n* = 52)	Lose (*n* = 65)	H	*p*	η^2^
	M ± SD	M ± SD	M ± SD			
Fouls	18.80 ± 4.74	18.75 ± 4.73	17.26 ± 4.54	3.008	0.222	0.016
Set pieces	28.68 ± 5.28	30.28 ± 5.43	29.36 ± 5.23	3.328	0.189	0.018
Set pieces with shots	4.13 ± 3.88	3.19 ± 2.23	2.75 ± 1.80	8.160	0.017 ^†^	0.045
Corners	5.15 ± 3.37	4.61 ± 3.04	3.63 ± 2.42	7.524	0.023 ^†^	0.041
Corners with shots	1.72 ± 1.46	1.30 ± 1.36	1.04 ± 1.17	8.170	0.017 ^†^	0.045
Penalties	0.37 ± 0.51	0.25 ± 0.46	0.15 ± 0.36	6.28	0.040 ^†^	0.034
Penalties converted	0.38 ± 0.52	0.15 ± 0.41	0.07 ± 0.26	19.080	0.001 ^†‡^	0.105
Free kicks	4.24 ± 12.16	3.92 ± 2.16	3.66 ± 2.25	7.618	0.022 ^‡^	0.042
Free kicks with shots	0.73 ± 0.95	0.94 ± 0.97	0.67 ± 0.85	2.689	0.261	0.014
Offsides	2.29 ± 5.78	1.82 ± 1.46	1.707 ± 1.32	0.511	0.774	0.002

Note: M ± SD—Mean ± Standard Deviation, H—Kruskal–Wallis H statistic, *p*—*p*-value, η^2^—Eta Squared. ^†^ Lose ≠ Win (*p* < 0.05)|^‡^ Draw ≠ Win (*p* < 0.05).

**Table 3:** Defensive play metrics and their impact on match outcomes (win, draw, lose).

Variable	Win (*n* = 65)	Draw (*n* = 52)	Lose (*n* = 65)	H	*p*	η^2^
	M ± SD	M ± SD	M ± SD			
Conceded Goals	0.46 ± 0.81	1.15 ± 0.82	2.09 ± 1.07	81.687	0.001 *^†‡^	0.451
Losses	105.98 ± 16.72	108.96 ± 15.23	108.40 ± 13.80	0.970	0.615	0.005
Losses Low	18.26 ± 12.77	18.37 ± 6.95	18.71 ± 7.90	1.242	0.537	0.006
Losses Medium	40.86 ± 12.05	41.31 ± 8.05	42.94 ± 9.43	1.232	0.540	0.006
Losses High	48.40 ± 11.04	49.29 ± 11.27	46.75 ± 13.64	1.294	0.523	0.007
Recoveries	78.85 ± 12.26	79.48 ± 10.54	75.38 ± 12.01	3.958	0.138	0.021
Recoveries Low	34.02 ± 10.15	35.04 ± 8.31	33.71 ± 6.36	0.338	0.844	0.001
Recoveries Medium	33.92 ± 7.97	34.31 ± 7.73	32.47 ± 9.17	3.195	0.202	0.017
Recoveries High	10.91 ± 6.11	10.13 ± 4.61	9.20 ± 4.06	2.077	0.354	0.011
Duels	226.92 ± 41.32	239.54 ± 26.38	230.28 ± 31.21	4.355	0.113	0.024
Duels won	113.42 ± 23.01	115.90 ± 16.81	111.72 ± 15.21	1.716	0.424	0.009
Duels won (%)	49.21 ± 7.35	48.40 ± 4.73	48.61 ± 3.07	0.210	0.900	0.001
Offensive duels	73.06 ± 14.39	74.87 ± 12.73	73.80 ± 13.67	0.211	0.900	0.001
Offensive duels won	31.17 ± 7.29	31.63 ± 7.34	31.25 ± 6.77	0.530	0.767	0.002
Defensive duels	73.14 ± 14.93	74.87 ± 12.73	73.82 ± 12.48	0.207	0.902	0.001
Defensive duels won	42.95 ± 10.20	43.23 ± 8.90	43.18 ± 9.21	0.044	0.978	0.001
Offensive duels won (%)	41.44 ± 5.75	42.22 ± 7.04	42.58 ± 6.49	1.367	0.505	0.007
Defensive duels won (%)	57.11 ± 6.74	57.78 ± 7.04	58.31 ± 5.64	1.440	0.487	0.007
Aerial duels	42.13 ± 11.70	46.12 ± 12.54	41.74 ± 11.47	3.710	0.156	0.020
Aerial duels won	20.98 ± 6.99	22.37 ± 7.63	19.95 ± 6.60	2.769	0.250	0.015
Aerial duels won (%)	49.24 ± 10.25	48.48 ± 10.02	47.67 ± 9.23	2.592	0.274	0.014
Sliding tackles	5.95 ± 4.06	5.35 ± 2.65	5.69 ± 2.69	0.496	0.780	0.002
Sliding tackles successful	2.58 ± 1.79	2.38 ± 1.66	2.58 ± 1.61	0.462	0.794	0.002
Sliding tackles successful (%)	46.45 ± 26.60	44.11 ± 26.85	44.14 ± 22.69	0.273	0.872	0.001
Interceptions	42.94 ± 14.35	41.25 ± 10.28	45.48 ± 10.08	4.671	0.097	0.025
Clearances	16.98 ± 8.32	17.52 ± 8.07	16.23 ± 7.42	0.562	0.755	0.003

Note: M ± SD—Mean ± Standard Deviation, H—Kruskal–Wallis H statistic, *p*—*p*-value, η^2^—Eta Squared. * Lose ≠ Draw (*p* < 0.05)|^†^ Lose ≠ Win (*p* < 0.05)|^‡^ Draw ≠ Win (*p* < 0.05).

**Table 4:** Binary Logistic Regression analysis results for the key predictors of match outcome (win/lose).

Variable						95% CI for Exp(B)	
	B	S.E.	Wald	*p*	Exp(B)	Lower	Upper
Shots on target	0.526	0.119	19.560	0.001 *	1.693	1.340	2.137
Losses (medium)	0.046	0.023	3.797	0.051	1.047	1.000	1.096
Counterattacks	0.270	0.117	5.305	0.021 *	1.310	1.041	1.648
Free kicks	−0.211	0.061	12.142	0.001 *	0.810	0.719	0.912
Penalties converted	1.953	0.492	15.745	0.001 *	7.051	2.687	18.504
Offensive duels won	−0.081	0.034	5.629	0.018 *	0.923	0.863	0.986
Back passes	0.275	0.102	7.225	0.007 *	1.317	1.077	1.610
Back passes accurate	−0.223	0.104	4.580	0.032 *	0.800	0.653	0.981
Smart passes	0.168	0.069	5.910	0.015 *	1.183	1.033	1.354
Constant	−7.133	1.999	12.731	0.001 *	0.001	-	-

* *p* < 0.05.

## Data Availability

Data available on request from the authors and the data presented in this study are available on request from the corresponding author due to (specify the reason for the restriction).
